# Prevalence and Risk Factors of Work‐Related Musculoskeletal Disorders Among Dockworkers: The Role of Psychosocial Stress and Physical Demands in an Iranian Port

**DOI:** 10.1002/hsr2.71840

**Published:** 2026-03-08

**Authors:** Abdolhamid Tajvar, Shokrollah Mohseni, Marzieh Kazempour

**Affiliations:** ^1^ Social Determinants in Health Promotion Research Center, Hormozgan Health Institute Hormozgan University of Medical Sciences Bandar Abbas Iran; ^2^ Department of Occupational Hygiene, Faculty of Health Hormozgan University of Medical Sciences Bandar Abbas Iran

**Keywords:** dock workers, job content questionnaire, musculoskeletal disorders, psychosocial factors

## Abstract

**Background and Aim:**

With the development of trade between countries, the transportation of goods has increased, and ports struggle to accommodate the demands of unloading and loading operations. Seaport workers may experience work‐related musculoskeletal disorders (WMSDs), which in many cases result in disabilities. Few studies have investigated the prevalence of work‐related musculoskeletal disorders (WMSDs) and associated psychological factors among dock workers in Iran. Existing findings indicate a high prevalence of WMSDs in these settings, primarily attributed to adverse working conditions such as repetitive movements, awkward postures, heavy lifting, and excessive physical strain. Moreover, there is a scarcity of research examining the impact of psychological factors on musculoskeletal disorders within this occupational group. This study investigated the relationship between work‐related psychological factors and job characteristics and WMSDs at the Port of Shahid Rajaee in Bandar Abbas, Iran, from December 2023 to April 2024.

**Methods:**

In this cross‐sectional study, all 351 dock workers were enrolled as research subjects. Dock workers in our study encompassed four distinct occupational roles with different physical and postural demands, including Trans Trainer Operators, Gantry Crane Operators, Tractor Drivers, and warehouse workers. To collect data, the Job Content Questionnaire (JCQ) and the Nordic Musculoskeletal Questionnaire (NMQ) were used. The sampling method used in this study was a census approach, encompassing all eligible workers at Sina port and Marine Service Development Company. Statistical analyses included descriptive statistics, independent *t*‐tests, Pearson's correlation coefficients, and one‐way analysis of variance (ANOVA).

**Results:**

The NMQ scores across the four groups were highest for the neck, shoulder, and upper back regions, with a mean score of 38.02, and lowest for the upper limb region, with a mean score of 10.40. Trans trainer and gantry crane operators and warehouse workers had significantly higher WMSDs in neck, shoulder, and upper back than tractor drivers (*p* < 0.001). Additionally, NMQ scores for the lower back were significantly correlated with the job type (*p* < 0.001), with warehouse workers having the highest MSD scores (22.85). Higher psychological job demands and psychosocial and physical stress were associated with more severe WMSDs for the workers. In addition, type of the job, age, work experience, marital status, and high job insecurity were significant factors influencing WMSDs.

**Conclusion:**

Work‐related psychosocial factors play an important role in the development of work‐related musculoskeletal disorders (WMSDs) among port workers. To mitigate these risks, it is essential to address psychological stress and job demands. Additionally, improving communication between port workers and management may contribute to a healthier and more supportive work environment.

## 1 Introduction

Dockworkers carry out various tasks essential to optimizing the operations of marine ports [1]. The onshore industry plays a vital role in international trade, reportedly accounting for approximately 80% of global transportation volume [2]. Containerization makes it possible to transport goods effectively both by sea and by land, hence container port terminals are key in sea‐land trade chains [3].

Workers on docks and in maritime industries are susceptible to various health issues, including work‐related musculoskeletal disorders (WMSDs) [4, 5, 6, 7]. For instance, a study reported dockworkers often face heavy workloads due to manual tasks [8]. The high prevalence of WMSDs among dockworkers is linked to working conditions, including heavy loads, repetitive tasks, and limited rest periods. These factors underscore the need for increased attention and preventive measures targeting this occupational group [4, 5].

At the port terminal, there are some special types of jobs, including gantry crane operators, truck drivers, and warehouse workers with high WMSD risk factors [2, 3]. In these types of jobs, improper posture, intense working conditions, repetitive tasks and movements, and psychological work pressure can be decisive in the occurrence of musculoskeletal disease [6, 7, 8].

WMSDs are among the most common occupational health issues, and globally were the main for work‐related disability in 2019 [9]. Approximately 2.3 million people worldwide die every year because of occupational injuries and work‐related diseases [10]. The International Labor Organization reported that work‐related diseases affect 160 million people annually [10]. Furthermore, more than a million workers experience work‐related injuries each year, often caused by repetitive motions, awkward postures, and excessive strain. According to the reports, WMSDs impose enormous costs on global healthcare systems [11]. Notably, musculoskeletal disorders occur at a higher prevalence and frequency in developing countries compared to other countries. In Iran, a ten‐year study conducted by Parno et al. showed that the prevalence of WMSDs is higher compared to similar countries, highlighting the need for in‐depth research and preventive solutions [11].

WMSDs are multi‐causal problems, with several factors that may affect their development [12]. The physical and individual factors that influence WMSDs include lifestyle, habits, age, sex, BMI, chronic disease, exercise, and smoking [11]. Psychological factors related to work and the work environment can also affect WMSDs [13, 14].

Dockworkers experience significant mental demands due to the need for sustained concentration and constant attention during their tasks [5]. Studies have shown that physical and psychological stress are the primary contributors to the majority of work‐related musculoskeletal disorders [15]. The most common method of assessing the psychological aspects of work is the job demand control model presented by Karasek et al. [16]. In this model, job stress is determined based on job demands, job control, and social support [16]. In the need‐control model, which considers the interaction between psychological needs and control, four major types of work are described: high strain (low control and high need), low strain (high control and low need), passive job (low control and low need), and active job (high need and high control) [17]. Studies using this model have shown that inappropriate conditions, such as high job demands, low levels of job control, and low social support at the workplace, decrease job satisfaction and increase the risk of cardiovascular disease and WMSDs [16, 18, 19].

Despite the critical role of onshore industries and docks, and the frequent reports of WMSDs, few studies have examined the prevalence of WMSDs in these sectors, particularly in Iran. Furthermore, few studies have investigated the combined effects of work‐related factors, including psychological and psychosocial variables, on musculoskeletal disorders [1, 9, 10]. Notably, a World Health Organization (WHO) document outlines a target to be achieved between 2015 and 2025, emphasizing the importance of addressing such occupational health issues [5]. Therefore, this study was conducted to investigate the prevalence of WMSDs and their relationship with psychological and work‐related parameters at one of the docks in southern Iran.

## 2 Materials and Methods

### 2.1 Study Design, Sample, and Procedure

This cross‐sectional study was conducted on male dock workers with four distinct occupational roles with different physical and postural demands, including trans trainer operator (*n* = 50), gantry crane operator (*n* = 50), tractor driver (*n* = 151), and warehouse workers (*n* = 100) at Sina port and marine service in Southern Iran. The data for this study were collected through direct, in‐person interviews with the dock worker. Using a census sampling method, all workers who met the following criteria were included in the study: at least 1 year of job experience, a high school diploma or higher educational qualification, no history of musculoskeletal disorders before employment, and a minimum score of 2 in at least one region on the Nordic Musculoskeletal Questionnaire (NMQ). Of the 386 workers at the Sina Port and Marine Services Development Company, 35 were excluded due to unwillingness to participate, the presence of systemic conditions affecting other body systems (e.g., neurological disorders, rheumatologic diseases, malignancies), or a history of joint replacement. Consequently, a total of 351 workers were included as study participants. To ensure data quality, the researchers reviewed all completed questionnaires and randomly rechecked a subset of responses for accuracy and consistency.

### 2.2 Measurements

Demographic and occupational information collected in this research included age, BMI, marriage, work experience, work hours, having a second job, shift work, and cigarette and hookah smoking status. Four job groups were included in this study: gantry crane operators, trans trainer operators, tractor drivers, and warehouse workers.

#### 2.2.1 Job Content Questionnaire (JCQ)

The job strain model was the basis of the JCQ. The Persian version of the JCQ was used in this study, which was validated and published by Choobineh et al. [20]. JCQ has five dimensions: job control, including nine items and contains the subscales of the skill discretion (which is control over the use of skills) and decision authority (which is control over various aspects of job performance), job psychological demands, which is evaluated by five questions and includes subscales of support from colleagues and support from supervisors, job physical demands, which are assessed by five questions and include the physical effort and isometric physical load subscales, and job social support and job insecurity, which are evaluated by 11 questions. All the questions are scored on a four‐point scale (strongly agree to strongly disagree).

#### 2.2.2 Nordic Musculoskeletal Questionnaire (NMQ)

In this study, the Persian version of the NMQ questionnaire used in this study is a validated instrument [21]. NMQ was originally used to assess the symptoms, duration, frequency, and intensity of pain in musculoskeletal regions of the body. A Likert scale ranging from 0 to 4 was employed for each body part, where “0” indicates no pain and “4” indicates severe pain. The scoring criteria for the NMQ were defined by the temporal occurrence of symptoms and their functional consequences. A score of 0 was assigned for no symptoms. A score of 1 was given for symptoms occurring solely within the preceding 12 months. Participants received a score of 2 if they reported symptoms in the last 12 months and the last 7 days, but without any associated disability. A score of 3 was allocated for symptoms within the last 12 months that caused disability. The highest score of 4 was assigned when symptoms were present in both the last 12 months and the last 7 days, and also caused disability. The global score, derived from the sum of all regional scores, had a potential range of 0–36 [21]. Pain intensity was used as the index for statistical analysis in this study. The internal consistency of the questionnaire was reported as 0.8, with Kappa values less than 0.7 and a significance level of *p* < 0.001 [22, 23].

According to a literature review [12, 24] and the characteristics of musculoskeletal disorders, the 11 regions of the body were classified into four regions [1]: shoulder, neck, and upper back region [2]; upper limb region (wrists, hands, and forearms) [3]; lower back region; and [4] lower limb region (hips, tights, knees, feet, and ankles). To calculate the percentage score of each area, the score of each categorized region was divided by the total score of the 11 regions and then multiplied by 100 [12].

#### 2.2.3 Data Analysis

Data analysis was performed using SPSS software, version 26.0. Both descriptive and inferential statistical methods were applied. Descriptive statistics included relative frequencies for categorical variables and means with standard deviations for continuous variables. Inferential analyses involved independent *t*‐tests, one‐way ANOVA, Pearson's correlation coefficients, and multivariate linear regression. The musculoskeletal disorder (MSD) score served as the dependent variable in the regression models, while job type, demographic characteristics, and work‐related psychological factors were included as independent variables. Independent *t*‐tests and one‐way ANOVA were employed to compare means across groups defined by categorical variables, including those with more than two categories. Pearson's correlation coefficient was used to evaluate relationships between continuous variables. Multivariate linear regression was conducted to identify factors associated with the severity of MSD symptoms [25]. A two‐sided *p* value of less than 0.05 was considered statistically significant.

The tables include several statistical terms and abbreviations commonly used in data analysis. The *t*‐distribution is employed in hypothesis testing and constructing confidence intervals for the mean of a single population or the difference between means of two populations. The F‐distribution is used similarly but focuses on hypothesis testing and confidence intervals for the ratio of variances between two populations. The term F/t represents the ratio of the F‐distribution to the t‐distribution. Standard deviation (SD) measures the variability or dispersion of data around the mean. Confidence intervals (CI) indicate the range within which the true population parameter is expected to lie, expressed as the estimated mean plus or minus the margin of error. The correlation coefficient (*r*) quantifies the strength and direction of the linear relationship between two continuous variables. Finally, the *p* value is a statistical measure used to evaluate the validity of a hypothesis based on observed data, with smaller values providing stronger evidence against the null hypothesis.

### 2.3 Ethical Consideration

Ethical approval for this study was obtained from the Research Center for Social Determinants of Health Promotion at Hormozgan University of Medical Sciences, Bandar Abbas, Iran. The certificate number is: IR.HUMS.REC.1402.249. Participants were informed that their personal information would be kept confidential and securely protected. The study's objectives were clearly explained, and participants were assured that the data collected would be used solely for research purposes. Additionally, it was emphasized that participation was voluntary, and individuals were free to withdraw from the study at any time without consequence.

## 3 Results

### 3.1 Demographic Characteristics, Job Background, and WMSDs

The participants had a mean work experience of 13.9 years (SD = 8.6), with only 6.6% (*n *= 23) holding a second job. The majority had a normal body mass index (BMI) and worked approximately 12 h per day. Among the subjects, 78% (*n *= 274) were married, and 32.5% (*n *= 114) had attained an academic education. Additionally, 23% (*n *= 81) reported cigarette smoking, while 28% (*n *= 132) used water pipes. The occupational distribution included 14% (*n *= 50) gantry crane operators, 14% (*n *= 50) trans trainer operators, 43% (*n *= 151) tractor drivers, and approximately 29% (*n *= 100) warehouse workers. The NMQ scores across the four groups were highest for the neck, shoulder, and upper back regions, with a mean score of 38.02, and lowest for the upper limb region, with a mean score of 10.40 (Table 1).

**Table 1:** Demographic characteristics, job background, and MSDs (*n* = 351).

Variable	Range	M ± SD	*n* (%)
Sex			
Female			—
Male			351 (100)
Age			
20–29	20–67	37.6 ± 7.8	52 (14.8)
30–39			162 (46.2)
40–49			111 (31.6)
> = 50			26 (7.4)
BMI			
18.5–24.9			198 (56.4)
25–29.9			146 (41.6)
> = 30			7 (2)
Work experience	1–44	13.9 ± 8.6	
1–9			132 (37.6)
10–19			145 (41.3)
> = 20			74 (21.1)
Marital status			
Single			77 (21.9)
Married			274 (78.1)
Education status			
Under diploma and diploma			237 (67.5)
Higher than a diploma			114 (32.5)
Second job status			
Yes			23 (6.6)
No			328 (93.4)
Status of cigarette			
No smoking			270 (76.9)
Sometimes (less than 1p/day)			81 (23.1)
Status of hookah			
No hookah			219 (62.4)
Every day			132 (27.6)
Type of job			
Trans trainer operator			50 (14.2)
Gantry crane operator			50 (14.2)
Trailer truck driver			151 (43)
Warehouse worker			100 (28.5)
WMSDs area			
Neck, shoulder, and upper back		38.02 ± 24.9	
Upper limb		10.4 ± 12.37	
Lower back		18.02 ± 14.3	
Lower limb		27.6 ± 18.4	

**Figure 1:**
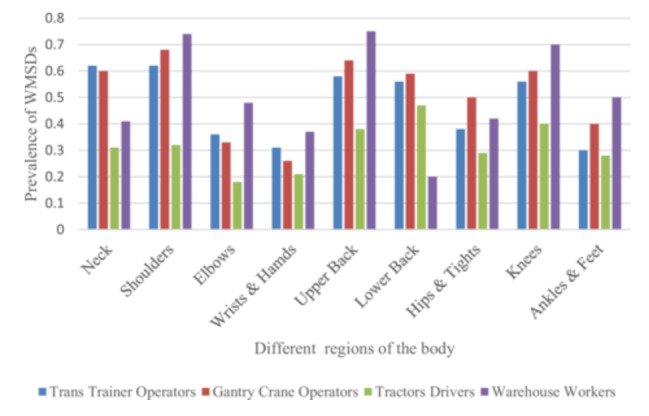
Prevalence of work‐related musculoskeletal disorders (WMSDs) in different body regions among various occupational groups at Sina Port. The Y‐axis shows the prevalence (%), calculated as the percentage of workers in each job group who reported a WMSD in the specified body region during the previous 12 months. Job groups are denoted by different colors.

Figure 1 illustrates that the lower back exhibited the highest prevalence of WMSDs across all job types, with the upper back, neck, and shoulder lagging considerably behind. Specifically, warehouse workers exhibited the highest prevalence of work‐related musculoskeletal disorders (WMSDs) in the upper back and shoulders, with rates of 0.75 (375/500) and 0.74 (372/500), respectively, and a knee prevalence of 0.70 (351/500). Gantry crane operators, trans trainer operators, and tractor drivers also showed high prevalence rates of lower back WMSDs at 0.59 (149/250), 0.56 (142/250), and 0.47 (361/755), respectively. Within these occupational groups, the shoulder, neck, and upper back regions were the next most affected areas. The least affected body parts among tractor drivers were the elbows and hands, with mean prevalence rates of 0.18 (140/755) and 0.21 (160/755), respectively.

### 3.2 Relationship Between WMSDs and Work‐Related Psychological Factors

Table 2 shows that job control was significantly related to WMSDs in the neck, shoulder, and upper back regions. Physical effort was significantly related to lower back WMSDs. Other univariate correlations of job content with WMSDs were insignificant.

**Table 2:** The correlation analyses of WMSDs and work‐related psychosocial factors (*n* = 351).

Variable	Mean	SD	Neck, shoulder, and upper back		Upper limb		Lower back		Lower limb	
			*r*	*p*	*r*	*p*	*r*	*p*	*r*	*p*
Psychological job demands	32.64	4.74	0.10**	0.04	0.16*	0.002	0.03	0.46	0.00	0.99
Physical job demands	13.11	3.63	0.110**	0.03	0.11**	0.03	0.15**	0.005	0.12**	0.02
Supervisor support	9.68	2.39	−0.16*	0.002	−0.12**	0.018	−0.02	0.59	−0.53	0.32
Coworker support	12.96	2.10	−0.04	0.41	−0.04	0.44	−0.13**	0.01	0.06	0.20
Social support	22.64	3.26	−0.15*	0.004	−0.11**	0.02	−0.10**	0.04	0.004	0.93
Physical effort	6.82	2.98	0.05	0.29	0.11**	0.03	0.2*	< 0.001	0.04	0.36
Ability to use skills	31.99	3.26	0.13**	0.011	0.16*	0.002	0.016	0.76	0.11**	0.03
Isometric physical load	6.28	1.85	0.12**	0.017	0.03	0.52	−0.02	0.61	0.16*	0.003
Job Insecurity	6.23	1.19	0.09	0.09	0.11**	0.03	0.13**	0.01	0.14**	0.007
Psychosocial stress	39.05	5.15	0.09	0.07	0.16*	0.002	0.06	0.25	0.013	0.80
Physical stress	20.88	2.11	0.14*	0.007	0.09	0.07	0.13**	0.011	0.16*	0.002

* Correlation is significant at the 0.01 level (2‐tailed).

** Correlation is significant at the 0.05 level (2‐tailed).

### 3.3 Associations of WMSDs With Job Background and Demographic Characteristics

According to Table 3, the results show that WMSDs in the neck, shoulder, and upper back were significantly correlated with the job type. Trans trainer, gantry crane operators, warehouse workers had significantly higher WMSDs in these body areas than tractor drivers. Additionally, NMQ scores for the lower back were significantly correlated with the job type, with warehouse workers having the highest MSD scores.

**Table 3:** Associations of WMSDs with job background and demographic characteristics (*n* = 351).

Variable	Neck, shoulder, and upper back			Upper limb			Lower back			Lower limb		
	M ± SD (%)	F/t	*p*	M ± SD (%)	F/t	*p*	M ± SD (%)	F/t	*p*	M ± SD (%)	F/t	*p*
Type of job												
Trans trainer operator	46.12 ± 23.47	13.24	< 0.001	12.25 ± 14.76	3.52	0.015	13.60 ± 9.90	7.213	< 0.001	27.73 ± 18.12	3.336	0.020
Gantry crane operator	48.73 ± 21.32			9.79 ± 12.00			13.83 ± 10.31			30.92 ± 19.19		
Tractors driver	29.18 ± 28.73			8.24 ± 12.07			17.66 ± 17.78			24.26 ± 21.06		
Warehouse	41.98 ± 15.00			13.03 ± 11.20			22.85 ± 10.06			30.94 ± 12.40		
Age												
20–29	34.41 ± 25.38	0.654	0.581	8.77 ± 11.65	0.408	0.747	19.93 ± 13.84	0.526	0.665	29.16 ± 18.42	2.852	0.037
30–39	37.78 ± 25.97			10.42 ± 13.62			17.77 ± 15.61			24.68 ± 18.80		
40–49	40.21 ± 24.71			11.04 ± 10.46			17.23 ± 13.07			29.84 ± 18.55		
> = 50	37.44 ± 17.78			10.80 ± 13.51			19.40 ± 11.94			33.18 ± 13.14		
BMI												
18.5–24.9	37.52 ± 23.96	0.131	0.877	11.12 ± 12.81	0.830	0.437	17.93 ± 14.43	0.311	0.733	27.23 ± 18.19	2.042	0.131
25–29.9	38.56 ± 26.40			9.54 ± 11.80			18.33 ± 14.13			27.46 ± 18.35		
> = 30	41.23 ± 24.06			7.89 ± 11.75			14.02 ± 15.93			41.50 ± 25.52		
Work experience												
1–9	37.90 ± 24.42	0.899	0.408	8.96 ± 11.95	2.681	0.070	18.89 ± 14.65	0.506	0.604	25.94 ± 18.52	2.568	0.078
10–19	39.71 ± 27.71			12.20 ± 13.28			17.16 ± 15.20			28.97 ± 18.45		
> = 20	34.94 ± 19.58			9.42 ± 10.88			18.14 ± 11.73			31.82 ± 17.96		
Marital status												
Single	34.70 ± 27.15	−1.324	0.186	9.97 ± 16.03	−0.347	0.729	20.32 ± 17.76	1.606	0.109	25.71 ± 19.92	−1.022	0.307
Married	38.96 ± 24.26			10.52 ± 11.17			17.37 ± 13.14			28.14 ± 18.03		
Education status												
Under diploma and diploma	36.80 ± 23.71	−1.32	0.185	10.62 ± 11.18	0.484	0.629	19.68 ± 14.06	3.191	0.002	28.42 ± 17.64	1.0185	0.237
Higher than diploma	40.57 ± 27.27			9.94 ± 14.59			14.55 ± 14.25			25.92 ± 20.03		
Second job status												
Yes	32.83 ± 27.38	−1.033	0.302	15.28 ± 18.52	1.963	0.050	13.85 ± 15.58	−1.446	0.149	27.64 ± 22.98	3.336	0.020
No	38.39 ± 24.77			10.06 ± 11.79			18.31 ± 14.19			27.60 ± 18.14		
Status of cigarette												
No smoking	38.22 ± 25.94	0.263	0.793	10.35 ± 12.81	−0.137	0.891	17.56 ± 14.21	−1.093	0.275	27.38 ± 18.86	−0.419	0.676
Sometimes (less than 1p/day)	37.38 ± 21.43			10.56 ± 10.86			19.54 ± 14.62			28.38 ± 17.14		
Status of hookah												
No hookah	36.48 ± 24.31	−1.465	0.136	10.38 ± 12.87	−0.044	0.965	17.04 ± 14.92	−1.648	0.10	28.91 ± 18.76	−0.910	0.363
Every day	40.58 ± 25.85			10.44 ± 11.55			19.63 ± 13.13			28.76 ± 17.97		

### 3.4 Prediction and Analysis of WMSDs Among Dock Workers

Using the enter method, we performed multiple linear regression analysis of the total WMSDs. Table 4 presents a regression model incorporating occupational background, significant demographic characteristics of dockworkers, and work‐related psychological factors.

**Table 4:** Hierarchical analysis of factors affecting WMSDs (*n* = 351).

Variable	Unstandardized coefficients B	Std. error	Standardized coefficients beta	*t*	Sig.	95.0% Confidence interval for B	
						Lower bound	Upper bound
Type of job							
Trans trainer operator	3.205	1.975	0.100	3.623	0.016	0.681	7.090
Gantry crane operator	7.332	1.899	0.229	3.861	< 0.001	3.596	11.068
Warehouse worker	12.625	2.477	0.508	5.097	< 0.001	7.752	17.498
Age							
30–39	0.278	1.754	0.009	0.158	0.874	−3.172	3.727
40–49	4.644	1.356	0.193	3.426	< 0.001	1.978	7.311
> = 50	5.181	2.325	0.121	2.229	0.026	0.609	9.754
BMI							
25–29.9	−0.514	1.027	−0.023	−0.501	0.617	−2.533	1.506
> = 30	1.463	3.528	0.018	0.415	0.679	−5.478	8.404
Work experience							
10–19	1.415	1.471	0.062	0.962	0.337	−1.478	4.308
> = 20	4.065	1.614	0.148	2.518	0.012	0.889	7.240
Marital status							
Married	3.943	1.445	0.146	2.728	0.007	1.100	6.787
Education status							
Higher than diploma	−1.820	1.411	−0.076	−1.290	0.198	−4.596	0.955
Second job status							
Yes	0.470	2.069	0.010	0.227	0.820	−3.601	4.541
Status of cigarette							
Sometimes (less than 1p/day)	0.690	1.189	0.026	0.580	0.562	−1.648	3.028
Status of hookah							
Every day	1.482	1.040	0.064	1.426	0.155	−0.563	3.527
Psychological job demands	4.151	1.176	1.757	3.531	< 0.001	1.839	6.464
Physical job demands	0.791	0.158	0.257	5.004	< 0.001	0.480	1.102
Supervisor support	−0.431	0.229	−0.092	−1.884	0.060	−0.881	0.019
Coworker support	−0.064	0.242	−0.012	−0.265	0.791	−0.539	0.411
Social support	−0.330	0.173	−0.096	−1.908	0.057	−0.670	0.010
Physical effort	1.531	0.428	0.408	3.574	< 0.001	0.688	2.374
Ability to use skills	0.245	0.173	0.071	1.421	0.156	−0.094	0.585
Isometric physical load	0.332	0.279	0.054	1.190	0.235	−0.216	0.880
Job insecurity	4.823	1.194	0.516	4.039	< 0.001	2.474	7.172
Psychosocial stress	4.031	1.152	1.853	3.499	< 0.001	1.765	6.298
Physical stress	1.224	0.363	0.231	3.374	< 0.001	0.510	1.938

*Note: R* ^2^ = 0.419.

The results show that demographic characteristics, job type, and age significantly affected WMSDs among dockworkers. Specifically, being a trans trainer operator (95% CI: 3.59–11.06, *p* < 0.001), a gantry crane operator (95% CI: 7.75–17.49, *p* < 0.001), and belonging to the 40–49‐year‐old age group (95% CI: 1.97–7.31, *p* < 0.001) were significantly associated with increased risk of WMSDs. These included psychological job demands (95% CI: 1.83–6.46, *p* < 0.001), physical job demands (95% CI: 0.48–1.10, *p* < 0.001), physical effort (95% CI: 0.68–2.37, *p* < 0.001), job insecurity (95% CI: 2.47–7.17, *p* < 0.001), psychosocial stress (95% CI: 1.76–6.29, *p* < 0.001), and physical stress (95% CI: 0.51–1.93, *p* < 0.001). Additionally, the regression model explained 41.9% of the variance in work‐related musculoskeletal disorders (WMSDs).

## 4 Discussion

This study aimed to investigate the prevalence of WMSDs and their relationship with psychological and work‐related parameters at one of the docks in southern Iran. One of the variables found to be correlated with WMSDs was the type of job held by the worker, and this was also found in studies of workers in Kuwait, Thailand, and Indonesia [26, 27, 28]. Compared to trailer truck drivers, gantry crane operators, transtainer operators, and warehouse workers showed significant increases in musculoskeletal disorder (MSD) scores of 7.3, 3.2, and 12.6 points, respectively.

**Figure 2:**
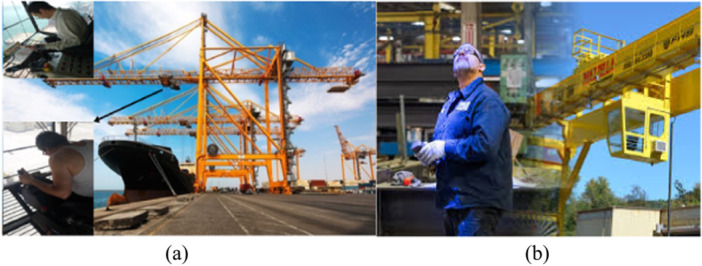
Sea port container cranes and their workers' postures: (a) gantry crane and (b) transtainer.

**Figure 3:**
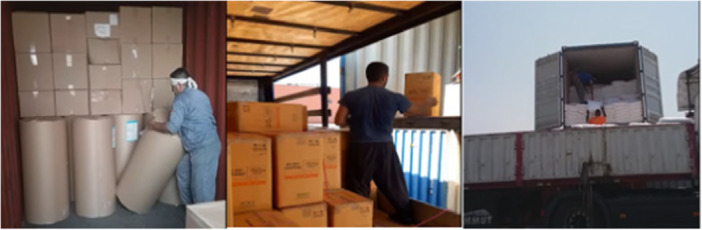
Awkward posture of warehouse workers.

In maritime shipping involving containerization, operators of gantry cranes and transtainers are key workers. This study showed that disorders in the neck and shoulders were the most common WMSDs in as many as 63% of port crane operator groups. The second most common regions afflicted by WMSDs, at almost 60%, were the upper and lower back areas. As shown in the Figure 2a, the reason for these musculoskeletal disorders in gantry operators is that the gantry control cabin is located 30–40 m above the ground level, and the operator should constantly lean forward and look downward in order to have better control over the load movement. This, in turn, results in a non‐neutral posture. In addition, the long continuous working hours which usually last more than 4 h are another contributing factor to these disorders [29, 30, 31]. Trans trainer operators also had the most WMSDs in the neck, shoulder, and the upper back. They perform precise container‐handling tasks to keep port operations moving efficiently (Figure 2b). However, the nature of their work exposes them to several ergonomic risk factors. Transtainer operation typically involves prolonged sitting in fixed, awkward postures, frequent neck flexion and rotation to monitor containers, and continuous upper‐limb micro‐movements when manipulating controls. Operators are also exposed to whole‐body vibration from machinery, which contributes significantly to spinal strain. All these risk factors could contribute to the development of MSDs in trans trainer operators [32, 33]. Warehouse workers had the most problems with the lower back, at 79%, and the second‐most problems (i.e., second‐highest MSD scores) for the upper back and shoulder, at 74.5%. They are required to perform tasks such as sorting, lifting, bending at the waist, moving and caring heavy loads, and other intense activities that affect their bodies over the long term (Figure 3), which can be the cause of the mentioned MSDs. Parallel to our findings, several researchers found very high ergonomic risk scores for these types of jobs, which can be dangerous, and they recommended that changes in work procedures be implemented as soon as possible [1, 29, 34].

The results of this research showed that older workers had higher Nordic musculoskeletal questionnaire scores for the lower limbs. It has been shown that the age‐groups of 40–49 years and 50 years and older had increased MSD scores of 4.6 and 5.1, respectively, in comparison with the younger age‐group. Previous studies also showed that older age was an important predictor of WMSDs [30, 31, 32]. This study showed that the workers with more than 20 years of job experience had a significant increase of four WMSDs compared with workers who had been on the job for fewer years. This result was parallel with the results of previous studies [7, 24, 25]. The reasons are age‐related degeneration of joints, reduced muscle strength, slower recovery, and longer exposure to occupational loads. In addition, this study showed that married workers had higher MSD scores, by as much as 3.9, than did single persons. The reason could be that married men in Iran have to make more efforts to meet their families' needs, and this may mean taking on additional work or tasks that could be associated with more WMSDs. The results of this research were consistent with those of Fazli et al. [33] and Aghilinejad et al. [35], who concluded that there is a significant association between marital status and the prevalence of WMSDs.

According to our findings, the level of psychosocial pressure in the study's job groups was high due to several factors, including repetitive tasks, low salary, conflicts with coworkers and supervisors, job insecurity, no promotion opportunity, and long working hours. The results indicated that psychological variables—including job demands, job insecurity, and psychosocial stress levels—were significantly associated with increases in MSD scores. This finding can be explained by the fact that psychological stress heightens muscle tension, diminishes physiological recovery, and promotes the accumulation of fatigue, thereby exacerbating musculoskeletal strain. Furthermore, workers who experience job insecurity often refrain from taking breaks and tend to accept heavier workloads, which further intensifies their physical and psychological burden. The results of the present study are consistent with those reported by Maëlys Clinchamps et al. [36]. In our analysis, a one‐unit increase in these psychological parameters was associated with a four‐unit increase in the musculoskeletal disorders score, underscoring the substantial impact of psychosocial factors on the development and severity of WMSDs. According to our findings, high levels of psychological demands, stress, and job insecurity were predictors of severe WMSDs. This result is in accordance with the job demand‐control model [36, 37, 38]. Furthermore, this study also revealed that the greater the physical effort, physical stress, and physical job demands, the higher the MSD score. When tasks require more force, the muscles and joints must work harder and this leads to higher muscle tension, increased joint compression, and faster fatigue which can result in MSDs development. These findings were similar to those of previous studies, which showed that there were significant correlations between psychological stress [12, 39, 40], physical effort [12, 39], and WMSDs in workers. Abdullah et al. [41] also studied the factors that caused lower back pain in workers. They found that in the stress‐related variables, one of the important factors that affected WMSDs was perceived general tension, which directly and indirectly influenced lower back pain. Our findings were consistent with the general theory that psychosocial occupational exposure influences health, and, as in previous studies, we found that psychosocial demands were important risk factors for WMSDs in workers [25, 42, 43].

## 5 Conclusion

The findings of this study indicate a significant association between job‐related psychological factors and work‐related musculoskeletal disorders (WMSDs) among port workers. Among these factors, psychological job demands, job insecurity, and overall psychosocial stress were the most influential. Additionally, WMSDs were more prevalent among workers exposed to high physical effort, physical stress, and physical job demands compared to those without such exposures. Based on these results, it is recommended that management implement pre‐employment training programs and consider reducing working hours. Moreover, enhancing communication channels and providing stronger managerial support may help alleviate worker stress and subsequently reduce the incidence of WMSDs.

### 5.1 Limitations and Suggestions

A limitation of our study was that it lacks a WMSDs risk assessment using a method like RULA to make a clearer picture of the awkward postures which lead to WMSDs. Future research should incorporate direct ergonomic assessments to quantify the specific physical risks identified in our study.

## Author Contributions

**Abdolhamid Tajvar:** project administration and resources. **Shokrolah Mohseni:** data curation, methodology, software, and validation. **Marzieh Kazempour:** conceptualization, supervision, and writing – original draft and editing.

## Ethics Statement

The study was approved by the ethics committee of Hormozgan University of Medical Sciences. The approved certificate number is: IR.HUMS.REC.1402.249. As directed by the Helsinki Declaration of 1964 and its amendments, ethical considerations were taken into account at every stage of the process. In addition, written informed consent was obtained from all participants before inclusion in the study. Participants were provided with detailed information about the study objectives, procedures, risks, and benefits, and were assured of confidentiality and their right to withdraw at any time without penalty.

## Conflicts of Interest

The authors declare no conflicts of interest.

## Transparency Statement

The corresponding author, Marzieh Kazempour, affirms that this manuscript is an honest, accurate, and transparent account of the study being reported; that no important aspects of the study have been omitted; and that any discrepancies from the study as planned (and, if relevant, registered) have been explained.

## Data Availability

The authors confirm that the data supporting the findings of this study are available from the corresponding author upon reasonable request.
